# Investigating causality in the association between 25(OH)D and schizophrenia

**DOI:** 10.1038/srep26496

**Published:** 2016-05-24

**Authors:** Amy E. Taylor, Stephen Burgess, Jennifer J. Ware, Suzanne H. Gage, J. Brent Richards, George Davey Smith, Marcus R. Munafò

**Affiliations:** 1 MRC Integrative Epidemiology Unit (IEU) at the University of Bristol, UK; 2 UK Centre for Tobacco and Alcohol Studies, University of Bristol, UK; 3 School of Experimental Psychology, University of Bristol, UK; 4 Department of Public Health and Primary Care, University of Cambridge, UK; 5 School of Social and Community Medicine, University of Bristol, UK; 6 Centre for Clinical Epidemiology, Lady Davis Institute for Medical Research, Jewish General Hospital, McGill University, Canada; 7 Department of Medicine, McGill University, Canada; 8 Department of Twin Research, King’s College London, UK

## Abstract

Vitamin D deficiency is associated with increased risk of schizophrenia. However, it is not known whether this association is causal or what the direction of causality is. We performed two sample bidirectional Mendelian randomization analysis using single nucleotide polymorphisms (SNPs) robustly associated with serum 25(OH)D to investigate the causal effect of 25(OH)D on risk of schizophrenia, and SNPs robustly associated with schizophrenia to investigate the causal effect of schizophrenia on 25(OH)D. We used summary data from genome-wide association studies and meta-analyses of schizophrenia and 25(OH)D to obtain betas and standard errors for the SNP-exposure and SNP-outcome associations. These were combined using inverse variance weighted fixed effects meta-analyses. In 34,241 schizophrenia cases and 45,604 controls, there was no clear evidence for a causal effect of 25(OH)D on schizophrenia risk. The odds ratio for schizophrenia per 10% increase in 25(OH)D conferred by the four 25(OH)D increasing SNPs was 0.992 (95% CI: 0.969 to 1.015). In up to 16,125 individuals with measured serum 25(OH)D, there was no clear evidence that genetic risk for schizophrenia causally lowers serum 25(OH)D. These findings suggest that associations between schizophrenia and serum 25(OH)D may not be causal. Therefore, vitamin D supplementation may not prevent schizophrenia.

Vitamin D deficiency has been linked with increased risk of a number of neuropsychiatric disorders, including schizophrenia^1^. Recent meta-analyses have shown that individuals with schizophrenia or psychotic disorders have lower levels of serum vitamin D and a higher prevalence of vitamin D deficiency relative to healthy controls, although not relative to individuals with other psychiatric disorders^2^,^3^. The association between low vitamin D and schizophrenia is supported by data on the prevalence of schizophrenia by latitude and by season of birth, which suggest that lower exposure to sunlight, which is required for the production of vitamin D, is associated with higher schizophrenia risk^1^,^4^. Vitamin D deficiency has also been proposed as a potential explanation for higher prevalence of schizophrenia amongst dark-skinned second generation migrants living in temperate climates^4^. It has been suggested that this observational association between lower vitamin D levels and increased schizophrenia risk may be due to a pathophysiological role of vitamin D in the aetiology of schizophrenia^3^. Furthermore, data from longitudinal studies provide some evidence that vitamin D supplementation or higher dietary vitamin D intake may reduce risk of psychosis or schizophrenia in some groups^5^,^6^. This has led to suggestions that randomised clinical trials (RCTs) are appropriate to explore the potential benefits of vitamin D supplementation for the prevention of schizophrenia^4^.

However, RCTs of vitamin D supplementation for schizophrenia prevention would be challenging and expensive, given that schizophrenia occurs at a relatively low frequency in the population. It is therefore important to establish with a high degree of certainty that vitamin D plays a causal role in the aetiology of schizophrenia, since if it does not an RCT will almost certainly fail. Unfortunately, establishing causality from observational data is notoriously problematic, due to well-described difficulties associated with confounding and reverse causality. Lower vitamin D levels in patients with schizophrenia may arise due to a pathophysiological causal effect of vitamin D levels on schizophrenia risk, or could be due to reverse causality, for example if individuals with schizophrenia are more likely to stay indoors and have reduced exposure to sunlight. Alternatively, the association between the two may not be causal at all but may arise due to confounding by independent risk factors. Possible confounders of this relationship include ethnicity, dietary factors, sedentary lifestyle, physical activity, body mass index, urban living and socioeconomic position, which have all been shown to be associated with both vitamin D levels and schizophrenia^4^,^7^,^8^,^9^,^10^,^11^,^12^,^13^.

Mendelian randomization (MR) is instrumental variable analysis that uses genetic variants as unconfounded proxies (i.e., instruments) for the exposure of interest. Due to the random nature of inheritance of genetic information, it can be assumed that we inherit each variant (for the most part) independently from other genetic variants, and independently from environmental factors, meaning that such variants are unlikely to be associated with potential confounding factors^14^. Moreover, because our genome is determined at conception, associations between genetic variants and outcomes cannot arise from reverse causation. To be a suitable instrument for a MR analysis, a genetic variant must be: 1) robustly associated with an exposure of interest, 2) not associated with potential confounding factors of the exposure-outcome association, 3) only associated with the outcome through the exposure of interest (i.e., not pleiotropic). If these assumptions are met, a genetic variant can be used in a MR experiment to test whether an observed association between and exposure and an outcome is likely to be causal^14^,^15^. Genomewide association studies (GWAS) have identified genetic variants (single nucleotide polymorphisms (SNPs)) which are robustly associated with circulating 25 hydroxyvitamin D (25(OH)D) (the most commonly measured biomarker of vitamin D) levels^16^,^17^. When combined in an allele score, these variants do not appear to associate with other lifestyle factors (with the exception of geographical region) which could confound the observed relationship between vitamin D and health outcomes^18^. Previous MR studies, using the serum 25(OH)D related genetic variants have provided evidence that serum 25(OH)D is unlikely to cause changes in body mass index^7^ or influence risk of diabetes, coronary artery disease or stroke^19^,^20^, but may causally influence multiple sclerosis^21^, all cause mortality^22^ and blood pressure^23^. More recently, variants robustly associated with schizophrenia have been identified in a GWAS conducted by the Psychiatric Genomics Consortium^24^. The associations of polygenic risk scores combining these variants with schizophrenia risk have been replicated in a number of independent populations^25^,^26^ and are now being used to investigate associations between schizophrenia and other phenotypes^26^,^27^.

We therefore took advantage of these findings and conducted a bidirectional Mendelian randomization analysis of the association between serum 25(OH)D levels and schizophrenia risk, to establish whether the observational association is likely to be causal. We conducted this analysis to examine two possibilities: 1) that serum 25(OH)D levels influence schizophrenia risk, and 2) that biological risk for schizophrenia influences serum 25(OH)D levels. In a conventional Mendelian randomization analysis, data on genotype, exposure and outcome from the same data set are analysed. However, obtaining complete exposure data may be difficult in some settings, due to high measurement costs or lack of appropriate biospecimens^28^. Two-sample Mendelian randomization refers to the use of data from separate samples, where data on genotype and the exposure of interest are available in one sample, and data on genotype and the outcome of interest available in the other^28^.

## Methods

### Study Design

**Figure 1:**
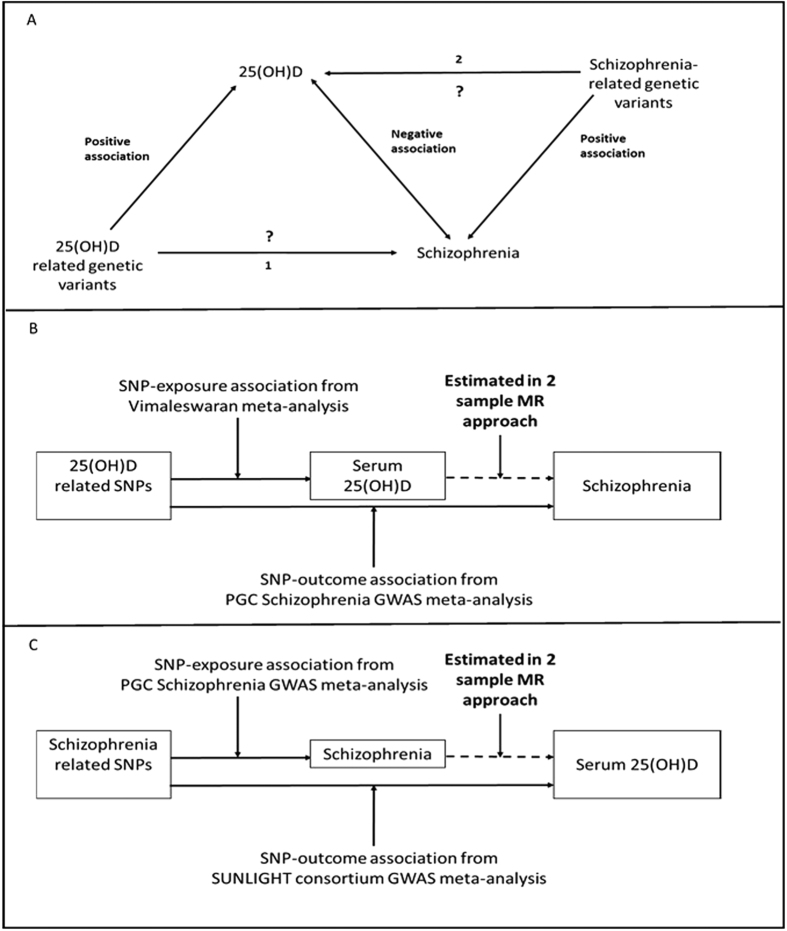
Bidirectional Mendelian randomization study of the association of 25(OH)D and schizophrenia. (**A**) Indicates direction of published genetic and observational associations. Analyses will provide estimates of (1) the causal effect of serum 25(OH)D on schizophrenia and (2) the causal effect of schizophrenia risk on serum 25(OH)D levels. (**B**) Shows data sources for investigating whether serum 25(OH)D causes schizophrenia. (**C**) Shows data sources for investigating whether biological risk for schizophrenia causally affects serum 25(OH)D.

We performed a two-sample bidirectional Mendelian randomization analysis. To investigate whether vitamin D causes schizophrenia we used single nucleotide polymorphisms (SNPs) associated with serum 25(OH)D levels as proxies for measured serum 25(OH)D in an analysis with schizophrenia as the outcome. To investigate whether schizophrenia may affect vitamin D levels, we used SNPs associated with schizophrenia as proxies for biological risk of schizophrenia in an analysis with serum 25(OH)D levels as the outcome. To obtain causal estimates from a Mendelian randomization analysis, the association (beta coefficients from linear regression or log odds ratios from logistic regression) between the SNPs and the outcome of interest (SNP-outcome association) are divided by the association of the SNP and the exposure of interest (SNP-exposure association). This is known as the Wald estimator^28^. For each analysis, estimates of the SNP-exposure and SNP-outcome associations were obtained from different sources. A schematic of the analysis is shown in Fig. 1.

### Ethical approval

This analysis is based on summary statistics obtained from previously published analyses and therefore we have not sought additional ethical approval. Each of these studies obtained appropriate ethical approval^7^,^16^,^24^.

### Vitamin D to Schizophrenia

To construct a genetic instrument for vitamin D, we used four genetic variants which demonstrated genome-wide significant associations with serum 25(OH)D levels in the SUNLIGHT GWAS meta-analysis (N = 16,125 in the discovery sample, N = 17,871 in the replication sample) conducted in individuals of European ancestry^16^. These variants were located in or close to the following genes: *GC* (rs2282679), *CYP2R1* (rs10741657), *DHCR7 (*rs12785878) and *CYP24A1 (*rs6013897). These genetic variants affect 25(OH)D levels through two distinct pathways (synthesis: *CYP2R1* and *DHCR7* and metabolism: *GC* and *CYP24A1*)^18^. Variants in or near three of these genes (*GC, CYP2R1, DHCR7*) have also reached genome-wide significance with serum 25(OH)D levels in a smaller GWAS^17^. None of these variants is in linkage disequilibrium (LD).

For the SNP-exposure association we used beta coefficients and standard errors from a meta-analysis (of up to 42,024 participants) conducted by Vimaleswaran and colleagues in individuals of European ancestry^7^ (see Table S1 in Supplementary material). These were reported as percentage change in serum 25(OH)D per effect allele. Effect sizes for associations with serum 25(OH)D were not available from the SUNLIGHT GWAS. This GWAS was conducted using a weighted z-score based approach^16^, meaning that contributing studies only shared z-scores (and not beta coefficients or standard errors) with the consortium.

For the SNP-outcome association, we used odds ratios and standard errors for the association of the vitamin D related variants with schizophrenia from the Schizophrenia GWAS meta-analysis conducted by the Psychiatric Genetics Consortium (PGC) (see Table S2 in Supplementary material)^24^. This is a case control study of 36,989 individuals with diagnosed schizophrenia and 113,075 controls (primarily of European ancestry). We used statistics from the GWAS discovery sample which comprised 34,241 cases and 45,604 controls and 3 family-based samples (1,235 parent affected-offspring trios). The summary statistics from the PCG GWAS meta-analysis are publicly available and can be downloaded at: http://www.med.unc.edu/pgc/downloads.

### Schizophrenia to Vitamin D

To construct a genetic instrument for schizophrenia, we used variants which reached genome-wide significance in the PGC schizophrenia GWAS, described above^24^. A total of 128 independent variants were identified (explaining around 3.4% of the variance in risk of schizophrenia), in 108 physically distinct genetic loci.

For the SNP-exposure association, we used odds ratios and standard errors for the association with schizophrenia from the discovery sample of the PGC^24^. Where SNPs from the PGC were not available in SUNLIGHT, we extracted proxy SNPs from SUNLIGHT that were in high LD with these variants (R^2^ > 0.9). A list of the proxies used is provided in Supplementary material (see Table S3 in Supplementary material). Of the 128 independent PGC GWAS significant variants, 81 SNPs were matched in SUNLIGHT (54 SNPs were matched directly, 27 were matched to proxy SNPs). Twelve (6 pairs) of the 81 SNPs included in the analysis were in low linkage disequilibrium with each other, with R^2^ values ranging from 0.03–0.20.

For the SNP-outcome association, we used the z-scores for the association of the schizophrenia-related SNPs with serum 25(OH)D from the SUNLIGHT consortium meta-analysis.

### Sample Overlap

We were unable to precisely determine the degree of sample overlap between studies contributing the gene-outcome and gene-exposure effect sizes. From the list of included studies, it is possible that up to 13% of the controls were in the Vimaleswaran meta-analysis and 12% of individuals in the Vimaleswaran meta-analysis were also PGC controls. Up to 6% of individuals included in the SUNLIGHT GWAS may have been PGC controls and 19% of PGC controls may have been in the SUNLIGHT GWAS. It is unlikely that there was any sample overlap between the vitamin D meta-analyses and the PGC cases.

### Statistical Analysis

#### Vitamin D to schizophrenia

SNP-exposure (serum 25(OH)D) and SNP-outcome (schizophrenia) associations for the four vitamin D related SNPs were combined in two different ways to estimate the association of vitamin D with schizophrenia. First, we used a fixed effects meta-analysis approach, which combines the ratio estimates (SNP-exposure divided by SNP-outcome) from individual variants using inverse variance weighting^29^. Standard errors for this method were approximated using the delta method^30^. Second, we used a likelihood based approach^31^. In both analyses, results are expressed as the odds ratio for schizophrenia per 10% increase in serum 25(OH)D. We combined estimates from all four vitamin D related SNPs in a single analysis. However, we also performed sensitivity analyses combining the estimates from SNPs in the synthesis (rs10741657 and rs12785878) and metabolism (rs2282679 and rs6013897) pathways separately. This has been done in previous Mendelian randomization studies investigating the causal effects of vitamin D^7^,^19^. In addition, we performed two further analyses: 1) excluding just the *DHCR7* SNP (rs12785878), which is strongly related to ancestry^18^ and 2) excluding just the *GC* SNP (rs2282679), as there is evidence that this SNP may have opposing effects on bioavailable vitamin D to its effects on 25(OH)D^32^ and that it could have potential pleiotropic effects, as the protein itself has been identified in cerebrospinal fluid^33^,^34^.

#### Schizophrenia to vitamin D

As associations of SNPs with serum 25(OH)D in SUNLIGHT were only available as test statistics (z-scores), we constructed beta coefficients and standard errors for the association of the schizophrenia-related SNPs with serum 25(OH)D from the z-scores, effect allele frequencies and sample size (see Table S4 in Supplementary material for formula). We then combined the SNP-exposure (schizophrenia) and SNP-outcome (serum 25(OH)D) associations for the 81 schizophrenia-related SNPs using the inverse variance weighted fixed effects meta-analysis approach and the likelihood based approach^29^. As the SNP-outcome associations in this analysis are constructed from z-scores, neither the beta coefficients for associations with serum 25(OH)D nor the effect sizes from the Mendelian randomization analysis have interpretable units. However, this analysis provides a direction of association and a P-value indicating the strength of the evidence for an association. To investigate the potential impact of correlated SNPs in this analysis, we performed a sensitivity analysis, excluding one SNP at random from the 6 pairs of SNPs in LD.

#### Detection of violations of the Mendelian randomization assumptions

A high degree of heterogeneity between estimates of the exposure-outcome association from SNPs included in the meta-analysis could indicate violation of the Mendelian randomization assumption that there is no pleiotropy (i.e., that all SNPs are only impacting on the outcome through their effect on the exposure)^35^. We therefore calculated Cochran’s Q and the I-squared statistic to estimate the degree of heterogeneity in the fixed effects meta-analysis. To further investigate potential bias due to pleiotropy, we performed Egger regression, as described by Bowden and colleagues^35^. The intercept from Egger regression is an estimate of the average pleiotropic effect of a SNP and so provides a test of directional pleiotropy, and the coefficient for the slope from Egger regression can provide a valid test of the causal effect estimate in Mendelian randomization analysis, even when one or more SNPs are pleiotropic.

In all analyses, confidence intervals were calculated as beta coefficients ± 1.96 * standard error. Analyses were performed in R (version 3.0.1) and Stata (version 11).

## Results

### Vitamin D to Schizophrenia

**Figure 2:**
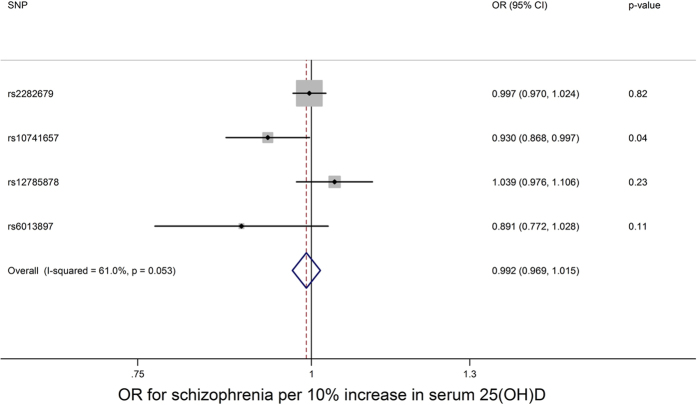
Mendelian randomization analysis of the effect of 25(OH)D on schizophrenia. OR = odds ratio. Results from fixed effects inverse variance weighted meta-analysis. Weights for each SNP in the meta-analysis are as follows: rs2282679: 72.6%, rs10741657: 11.2%, rs12785878: 13.6%, rs6013897: 2.6%.

Using genetic variants related to serum 25(OH)D levels as instruments for measured serum 25(OH)D levels, there was no clear evidence from the inverse variance weighted fixed effects meta-analysis for a causal effect of serum 25(OH)D levels on schizophrenia (OR for schizophrenia per 10 percent increase in serum 25(OH)D: 0.992, 95% CI: 0.969 to 1.015) (see Fig. 2). The likelihood method produced a similar result (OR: 0.991, 95% CI: 0.969 to 1.015).

There was some evidence for heterogeneity in the analysis (I^2^ = 61%, 95% CI: 0, 87), indicating that the SNPs did not demonstrate associations with schizophrenia consistent with the magnitudes of their effect on serum 25(OH)D. However, in Egger regression, there was no clear evidence for directional pleiotropy (Intercept: 0.982 (95% CI: 0.959, 1.005). There was no clear evidence that the causal effect estimate from Egger regression differed from the estimate we obtained from the inverse variance weighted and likelihood approaches (OR: 1.023, 95% CI: 0.977 to 1.072).

There was no clear evidence for causal effects of serum 25(OH)D on schizophrenia in the analyses using just the SNPs involved in 25(OH)D synthesis (OR from fixed effects meta-analysis: 0.988, 95% CI: 0.944 to 1.035) or in 25(OH)D metabolism (OR from fixed effects meta-analysis: 0.993, 95% CI: 0.967 to 1.020) (see Figs S1 and S2 in Supplementary material).

### Schizophrenia to Vitamin D

**Figure 3:**
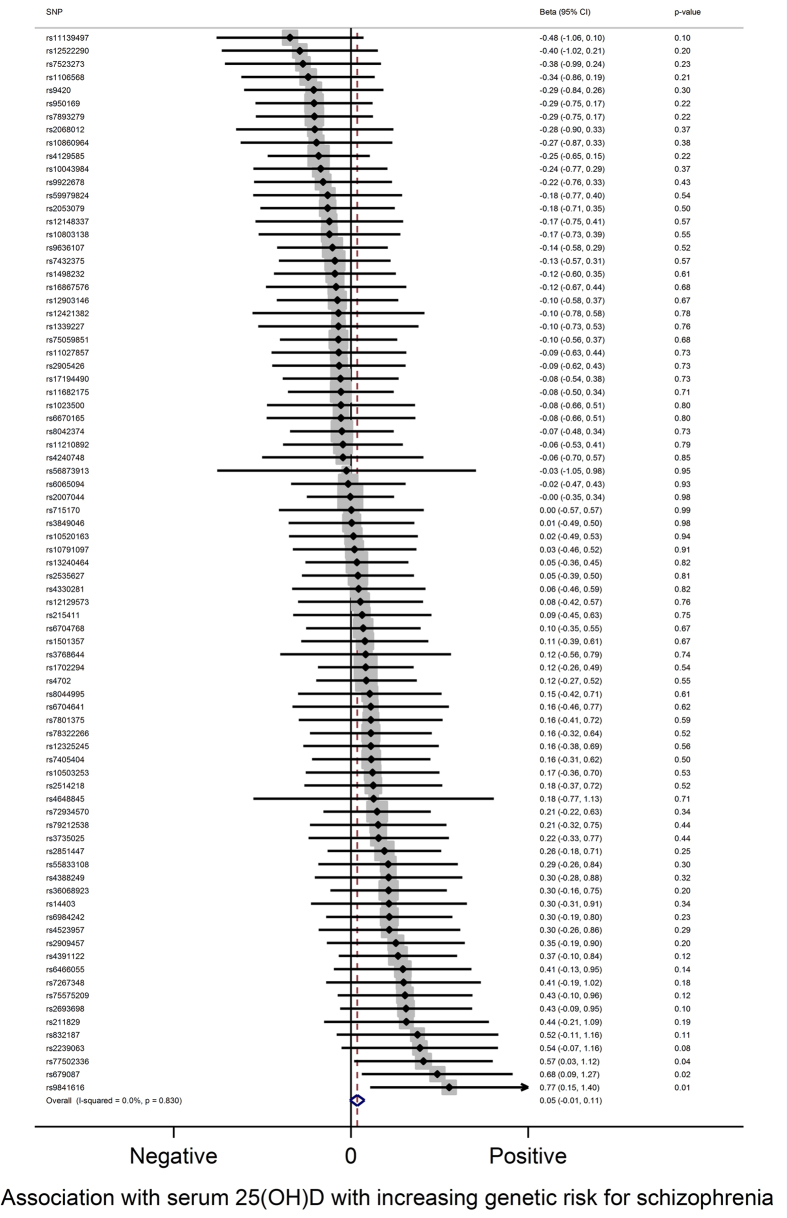
Mendelian randomization analysis of the effect of genetic risk for schizophrenia on serum 25(OH)D levels. Results from fixed effects inverse variance weighted meta-analysis. Weights for individuals SNPs ranged from 0.4 to 2.8%. Overall effect is in the positive direction (higher genetic risk for schizophrenia associated with higher 25(OH)D). Magnitude of effects from this analysis are not interpretable.

The analysis using genetic variants related to schizophrenia as instruments for risk for schizophrenia indicated only suggestive evidence for a causal effect of schizophrenia on vitamin D levels (P-value = 0.08 in the fixed effects meta-analysis). The effect was in the direction of higher genetic risk for schizophrenia increasing serum 25(OH)D levels (see Fig. 3). The likelihood approach produced similar results (positive direction of effect, P-value = 0.05). Excluding one of each SNP pair in LD slightly weakened the evidence for an effect (P-value from fixed effects meta-analysis = 0.14), but the direction of effect remained the same.

There was no evidence for heterogeneity in this analysis (I^2^ = 0%) and no clear evidence for directional pleiotropy (P-value = 0.79) or a causal effect (P-value = 0.54) from Egger regression. The full output from the fixed effect meta-analysis, the likelihood approach and Egger regression are presented in Supplementary material (Table S5).

## Discussion

Although there are strong observational associations between vitamin D deficiency and schizophrenia, our Mendelian randomization results do not provide any clear evidence that serum 25(OH)D levels play a causal role in schizophrenia, or that genetic risk for schizophrenia (as indexed by a schizophrenia polygenic risk score) causally lowers serum 25(OH)D levels. These findings would suggest that observational associations between serum 25(OH)D levels and schizophrenia risk could be explained by confounding due to lifestyle factors and health behaviours. Lack of evidence for a causal role of serum 25(OH)D in schizophrenia indicates that vitamin D supplementation may not be effective for prevention of schizophrenia.

We investigated the potential causal relationship between serum 25(OH)D and schizophrenia in the largest genetic study of schizophrenia published to date^24^. Using genetic markers related to serum 25(OH)D levels in a Mendelian randomization approach removes the possibility of reverse causality in this analysis and is likely to minimise confounding due to other lifestyle factors. As data on serum 25(OH)D levels are not available in the PGC, we have assumed that the vitamin D related genetic variants are associated with serum 25(OH)D levels in the schizophrenia cases and controls of the PGC consortium and that the relationship between serum 25(OH)D levels and schizophrenia risk is linear. Given the relatively well described function of the vitamin D genetic variants, which are located in or close to genes coding for proteins in vitamin D synthesis and metabolism pathways^18^, and the replication of these variants with serum 25(OH)D in additional populations^7^,^19^,^20^, we think that this first assumption is likely to be valid. With regards to the second assumption, it is possible that schizophrenia risk is only increased amongst individuals with 25(OH)D deficiency; if this is the case, the test for a causal effect of 25(OH)D from the MR analysis would still be a valid test of a causal link between 25(OH)D deficiency and schizophrenia. However, our power to detect threshold effects would be lower, particularly if prevalence of 25(OH)D deficiency is very low in the schizophrenia GWAS sample. There is evidence that the variants we have used are associated with likelihood of being 25(OH)D deficient^16^ and prevalence of 25(OH)D deficiency in European populations is reported to be reasonably high. For example, the average year-round prevalence of serum 25(OH)D below 25 nmol/L in adults in the UK (2008–2012) was reported to be 24% in males and 22% in females^36^. However, we cannot completely rule out the possibility of a threshold effect from the results of these analyses. For similar reasons, we must be cautious in generalising these results to populations of non-European ancestry, which have been shown to have higher prevalence of vitamin D deficiency and schizophrenia (as well as other mental health conditions)^4^,^37^,^38^.

Mendelian randomization is potentially an efficient method to establish or refute causality with respect to known observational associations. Indeed, it has been argued that the primary value of this approach will be to identify which observational associations are *not* likely to be causal, and thereby prevent investment in clinical trials of interventions which are likely to be unsuccessful^39^. Our results would appear to align with this objective, and suggest that clinical trials aimed at increasing serum 25(OH)D to prevent schizophrenia are not warranted. We assume that the 25(OH)D related genetic variants affect concentrations of serum 25(OH)D throughout the life-course. Whilst these results are therefore consistent with no causal effect of life-long lower serum 25(OH)D levels on schizophrenia risk, we may not be able to rule out there being a specific critical exposure period where low vitamin D increases risk of schizophrenia. For example, maternal 25(OH)D status during pregnancy, has been identified as a possible risk factor for schizophrenia^40^,^41^. The 25(OH)D SNPs should be associated with maternal serum 25(OH)D levels of the individuals included in this analysis, but only half as strongly. Furthermore, as our analysis was conducted in a case control sample of schizophrenia, we cannot use these data to make inferences about the potential impact of vitamin D supplementation in individuals already diagnosed with schizophrenia as an intervention for reducing symptoms^3^.

Well powered studies are required to have confidence that lack of evidence for causal effects in Mendelian randomization studies reflect true null associations^39^. Along with sample size, the strength of the association between the genetic variant and the exposure is a key determinant of power in Mendelian randomization analysis. Whilst the genetic variants used in this analysis only explain a small proportion of the variation in serum 25(OH)D levels (3.6% in the study by Ye and colleagues^19^), the increase in serum 25(OH)D conferred by each copy of the major allele of the *GC* SNP (rs2282579) has been shown to be roughly equivalent to taking a daily vitamin D supplement in some populations^16^. Therefore, failure of the combined 25(OH)D related genetic variants to demonstrate clear evidence of an association with increased risk of schizophrenia in the largest genetic case control sample of schizophrenia available to date enables us to be reasonably confident that 25(OH)D levels are not a major contributing factor for schizophrenia in European populations.

The analysis presented relates to serum 25(OH)D levels, the major circulating metabolite of vitamin D, which is a precursor to the active form (1, 25- dihydroxyvitamin D_3_)^18^. Therefore, whilst serum 25(OH)D is observationally very strongly associated with schizophrenia risk, we must exercise some caution in drawing conclusions regarding the role of biologically active vitamin D^19^. There is some evidence that one of the metabolism variants associated with increased serum 25(OH)D levels (rs2282679) may in fact reduce the bioavailability of vitamin D^19^,^20^,^32^. However, when we excluded this variant from the meta-analysis, there was no clear evidence for any association of serum 25(OH)D with schizophrenia (see Fig. S3 in Supplementary material). Furthermore, the SNP scores based on genetic variants involved in synthesis and metabolism also failed to provide convincing evidence for an effect of serum 25(OH)D on schizophrenia.

We also investigated the potential causal impact of genetic risk for schizophrenia on serum 25(OH)D, using genetic variants which have been identified to increase risk of schizophrenia^24^. Whilst there was some weak evidence for an association of the risk score with serum 25(OH)D levels, it was in the direction of schizophrenia *increasing* serum 25(OH)D, which is opposite to what is seen observationally. Therefore, this analysis does not provide convincing evidence that genetic risk for schizophrenia decreases serum 25(OH)D levels. In this analysis, the gene-exposure associations for the schizophrenia related genetic variants were derived from a case control sample of individuals with and without schizophrenia, but here we have applied them the studies to contributing to the SUNLIGHT consortium (largely comprising general population samples), where schizophrenia prevalence is likely to be low. There is evidence that schizophrenia symptoms exist on a continuum^42^,^43^,^44^, although this is debated^44^,^45^. If this is indeed the case, genetic risk for schizophrenia will likely influence schizophrenia-like symptoms in individuals without diagnosed schizophrenia. However, we are assuming in our analysis that this genetic risk on schizophrenic traits would be sufficiently strong to alter lifestyle in a way that could impact upon serum vitamin D in individuals without severe disease. Furthermore, we are assuming that this genetic risk would impact upon lifestyle in the same direction in the general population as in those with severe disease (i.e., that if individuals with severe disease have lower exposure to sunlight, higher genetic risk in individuals without clinical disease is also associated with lower exposure to sunlight). We should therefore treat the results of our analysis of schizophrenia risk on vitamin D with some caution.

There are some general limitations to Mendelian randomization that should also be considered when interpreting these results. First, we were not able to exclude the possibility of sample overlap in our analysis. With a small overlap, as it most likely the case here, it is likely that any bias would be conservative, and would not lead to a false positive finding^28^. Second, we cannot completely rule out population stratification and pleiotropy as sources of bias in these analyses. However, restriction of the data included to almost exclusively European individuals as well as adjustment for principal components within the contributing studies^7^,^16^,^24^ is likely to protect against spurious findings due to population stratification. Furthermore, removal of the *DHCR7* SNP (which is strongly associated with ancestry) from the analysis did not appear to influence the results (see Fig. S4 in Supplementary material). In addition, the results of Egger regression (which should be less subject to bias than inverse variance weighted meta-analysis) did not provide clear support for the existence of directional pleiotropy or for a non-null causal effect. However, Egger regression has much lower power to detect causal effects than inverse variance weighted meta-analysis, and the power of Egger regression to detect pleiotropy decreases with decreasing numbers of genetic variants^35^.

In conclusion, our findings, which should be subject to lower bias from potential confounding by lifestyle factors than conventional epidemiological studies, do not provide evidence that vitamin D levels causally affect schizophrenia. This finding is valuable as it suggests that vitamin D supplementation in the general population is unlikely to have large effects on schizophrenia risk, although we cannot completely rule out the possibility that deficient individuals could be at higher risk. Two-sample Mendelian randomization analysis using summary data is an efficient strategy for investigating potential causal links in the absence of large studies with genetic data, vitamin D and schizophrenia. Follow up of these findings in such studies would be beneficial in the future for better characterising the nature of the vitamin D and schizophrenia associations. Furthermore, similar analyses in populations of different ethnicities, who are more at risk of vitamin D deficiency and schizophrenia would help to clarify whether there may be subgroups who could benefit from supplementation.

## Additional Information

**How to cite this article**: Taylor, A. E. *et al.* Investigating causality in the association between 25(OH)D and schizophrenia. *Sci. Rep.* **6**, 26496; doi: 10.1038/srep26496 (2016).

## Supplementary Material

Supplementary Information
