# Supplementary material for: Investigating causality in the association between 25(OH)D and schizophrenia
Source: Sci Rep. 2016 May 24;6:26496. doi: 10.1038/srep26496 (PMC4877705; doi:10.1038/srep26496)
Supplement: Supplementary Information [file srep26496-s1.pdf]

Investigating causality in the association between 25(OH)D and schizophrenia

Amy E. Taylor PhD[1,2,3], Stephen Burgess PhD[1,4], Jennifer J. Ware PhD[1,2,5], Suzanne H. Gage PhD[1,2,3], SUNLIGHT consortium, J. Brent. Richards PhD [6,7,8], George Davey Smith MD [1,5], Marcus R. Munafò PhD[1,2,3]

1. MRC Integrative Epidemiology Unit (IEU) at the University of Bristol, UK
2. UK Centre for Tobacco and Alcohol Studies, University of Bristol, UK
3. School of Experimental Psychology, University of Bristol, UK
4. Department of Public Health and Primary Care, University of Cambridge, UK
5. School of Social and Community Medicine, University of Bristol, UK
6. Centre for Clinical Epidemiology, Lady Davis Institute for Medical Research, Jewish General Hospital, McGill University, Canada
7. Department of Medicine, McGill University, Canada
8. Department of Twin Research, King's College London, UK

Table S1. Association of vitamin D related genetic variants with 25(OH)D

SNP	Chr	Gene	Pathway	Alleles (minor/major)	Minor allele frequency	Effect per minor allele (% change in 25(OH)D)*	Standard error
rs2282679	4	*GC*	metabolism	G/T	0.26	-8.45	0.31
rs10741657	11	*CYP2R1*	synthesis	A/G	0.40	3.12	0.29
rs12785878	11	*DHCR7*	synthesis	G/T	0.22	-3.70	0.30
rs6013897	20	*CYP24A1*	metabolism	A/T	0.20	-1.85	0.33

SE: standard error; *Effect sizes and standard errors taken from Vimaleswaran et al. [1]

1 Vimaleswaran, K. S. *et al.* Causal relationship between obesity and vitamin D status: bi-directional Mendelian randomization analysis of multiple cohorts. *PLoS medicine* **10**, e1001383, doi:10.1371/journal.pmed.1001383 (2013).

Table S2. Association of vitamin D related genetic variants with schizophrenia in the PGC Schizophrenia GWAS

SNP	Chr	Gene	Effect allele	OR for schizophrenia per effect allele	Standard Error
rs2282679	4	*GC*	T	0.9973	0.0117
rs10741657	11	*CYP2R1*	A	0.97775	0.0108
rs12785878	11	*DHCR7*	T	1.0143	0.0118
rs6013897	20	*CYP24A1*	A	1.02163	0.0130

Data available to download from: http://www.med.unc.edu/pgc/downloads

Table S3. Associations of schizophrenia related SNPs included in the analysis with schizophrenia in the PGC

SNP	Chromosome	A1A2[1]	EAF[2]	Odds ratio	Standard error
rs4648845	1	TC	0.533	1.07144	0.0117
rs1498232	1	TC	0.311	1.0724	0.0115
rs11210892	1	AG	0.659	0.93286	0.0112
rs12129573	1	AC	0.377	1.07186	0.011
rs1702294	1	TC	0.175	0.89074	0.0137
rs6670165	1	TC	0.196	1.0739	0.0135
rs7523273	1	AG	0.695	1.06194	0.0115
rs10803138	1	AG	0.232	0.93165	0.0126
rs14403	1	TC	0.207	0.93454	0.0128
rs11682175	2	TC	0.52	0.92849	0.0106
rs75575209	2	AT	0.904	0.89583	0.0192
rs3768644	2	AG	0.0967	0.91037	0.0178
rs2909457	2	AG	0.568	0.94271	0.0108
rs59979824	2	AC	0.322	0.93576	0.0116
rs6704641	2	AG	0.819	1.07918	0.0138
rs6704768	2	AG	0.54	0.92886	0.0106
rs17194490	3	TG	0.169	1.10197	0.0148
rs4330281	3	TC	0.479	0.9428	0.0108
rs2535627	3	TC	0.545	1.07272	0.0106
rs832187	3	TC	0.607	0.94111	0.0109
rs7432375	3	AG	0.421	0.93109	0.0109
rs9841616	3	AT	0.158	0.92247	0.0143
rs215411	4	AT	0.331	1.06695	0.0114
rs10520163	4	TC	0.493	1.06279	0.0106
rs1106568	4	AG	0.747	0.93295	0.0122
rs1501357	5	TC	0.794	0.92635	0.0134
rs4391122	5	AG	0.505	0.92413	0.0107
rs16867576	5	AG	0.889	1.10054	0.0169
rs4388249	5	TC	0.212	1.07466	0.0135
rs10043984	5	TC	0.266	1.0694	0.012
rs3849046	5	TC	0.542	1.06471	0.0107
rs79212538	5	TG	0.0512	1.15165	0.0257
rs12522290	5	CG	0.84	1.08556	0.0147
rs1339227	6	TC	0.347	0.94186	0.0111
rs6466055	7	AC	0.35	1.06833	0.0111
rs211829	7	TC	0.641	1.05665	0.011
rs13240464	7	TC	0.667	1.08394	0.0112
rs7801375	7	AG	0.146	0.92035	0.0148
rs3735025	7	TC	0.657	1.06577	0.011
rs10503253	8	AC	0.223	1.07347	0.0127
rs6984242	8	AG	0.586	0.93763	0.0107

rs36068923	8	AG	0.787	0.91943	0.013
rs4129585	8	AC	0.447	1.08156	0.0107
rs11139497	9	AT	0.346	1.07037	0.0115
rs7893279	10	TG	0.899	1.11974	0.0171
rs55833108	10	TG	0.208	1.07842	0.0133
rs11027857	11	AG	0.515	1.0646	0.0106
rs9420	11	AG	0.327	1.0629	0.0113
rs12421382	11	TC	0.318	0.94261	0.0113
rs2514218	11	TC	0.31	0.93034	0.0116
rs77502336	11	CG	0.337	1.07079	0.0114
rs10791097	11	TG	0.479	1.0767	0.0106
rs75059851	11	AG	0.812	1.09615	0.0135
rs2007044	12	AG	0.602	0.91247	0.0108
rs2239063	12	AC	0.729	1.07144	0.0118
rs679087	12	AC	0.324	0.94073	0.0113
rs4240748	12	CG	0.358	0.94261	0.0111
rs10860964	12	TC	0.65	1.06056	0.011
rs2851447	12	CG	0.723	0.91302	0.0119
rs2068012	14	TC	0.76	0.93333	0.0126
rs2693698	14	AG	0.412	0.93894	0.0111
rs12903146	15	AG	0.544	1.06695	0.0106
rs12148337	15	TC	0.478	1.05908	0.0105
rs8042374	15	AG	0.75	1.09144	0.0124
rs950169	15	TC	0.247	0.92386	0.0122
rs4702	15	AG	0.547	0.92459	0.0112
rs9922678	16	AG	0.299	1.06962	0.0116
rs7405404	16	TC	0.238	1.08145	0.0125
rs12325245	16	AT	0.849	0.91631	0.0153
rs8044995	16	AG	0.173	1.0808	0.0141
rs4523957	17	TG	0.642	1.07069	0.0112
rs78322266	18	TG	0.0345	1.19363	0.031
rs9636107	18	AG	0.49	0.92663	0.0107
rs72934570	18	TC	0.0701	0.86528	0.0208
rs715170	18	TC	0.261	0.93594	0.012
rs2905426	19	TG	0.611	0.93725	0.0112
rs2053079	19	AG	0.755	0.92932	0.0124
rs56873913	19	TG	0.775	1.0693	0.0129
rs6065094	20	AG	0.307	0.92849	0.0113
rs7267348	20	TC	0.741	0.93782	0.0121
rs1023500	22	TC	0.817	1.07638	0.0135

1. A1 is the effect allele; 2. EAF: effect allele frequency.

Table S4. Associations of schizophrenia related SNPs included in the analysis with vitamin D levels in SUNLIGHT

SNP	Proxy[1]	R^2	N (SUNLIGHT)	p-value	z-score[3]	EAF[4]	Beta[5]	Standard error[6]
rs4648845			3627	0.7108	0.371	0.4789	0.0123	0.0332
rs1498232	rs267700	1	16100	0.6082	-0.513	0.2978	-0.0088	0.0172
rs11210892			15951	0.7922	0.263	0.6705	0.0044	0.0168
rs12129573	rs12144370	0.965	13989	0.7614	0.304	0.3847	0.0053	0.0174
rs1702294	rs2660304	1	14028	0.5428	-0.609	0.1863	-0.0132	0.0217
rs6670165			14042	0.7954	-0.259	0.1908	-0.0056	0.0215
rs7523273	rs7541230	1	14039	0.2156	-1.238	0.6757	-0.0223	0.0180
rs10803138			13978	0.5527	0.594	0.253	0.0116	0.0195
rs14403	rs884328	0.913	14039	0.3299	-0.974	0.2253	-0.0197	0.0202
rs11682175			16121	0.7103	0.371	0.5193	0.0058	0.0158
rs75575209	rs17049152	1	14045	0.1016	-1.637	0.8972	-0.0455	0.0278
rs3768644	rs3768641	0.915	11060	0.7347	-0.339	0.0975	-0.0109	0.0321
rs2909457	rs4664442	0.934	16081	0.1886	-1.315	0.5374	-0.0208	0.0158
rs59979824	rs12619354	0.903	11714	0.5366	0.618	0.3675	0.0118	0.0192
rs6704641			11829	0.619	0.497	0.8089	0.0116	0.0234
rs6704768			13945	0.6705	-0.426	0.5682	-0.0073	0.0171
rs17194490			13970	0.7308	-0.344	0.1695	-0.0078	0.0226
rs4330281	rs4243834	0.967	16096	0.8168	-0.232	0.4555	-0.0037	0.0158
rs2535627			16115	0.8083	0.243	0.5367	0.0038	0.0158
rs832187			11833	0.09064	-1.692	0.637	-0.0323	0.0191
rs7432375			16100	0.5728	0.564	0.3908	0.0091	0.0162
rs9841616			14048	0.006996	-2.697	0.1764	-0.0597	0.0221
rs215411			14025	0.7449	0.325	0.3215	0.0059	0.0181
rs10520163			16123	0.9361	0.08	0.4982	0.0013	0.0158
rs1106568			16061	0.1966	1.291	0.7577	0.0238	0.0184
rs1501357			16120	0.6649	-0.433	0.8174	-0.0088	0.0204
rs4391122			11829	0.1111	-1.593	0.5151	-0.0293	0.0184
rs16867576			13935	0.6808	-0.411	0.8715	-0.0104	0.0253
rs4388249			13652	0.3069	1.022	0.157	0.0240	0.0235
rs10043984	rs3798149	1	16121	0.3689	-0.899	0.2707	-0.0159	0.0177
rs3849046			16037	0.9818	0.023	0.5347	0.0004	0.0158
rs79212538	rs4133347	1	13995	0.4312	0.787	0.0459	0.0318	0.0404
rs12522290			11896	0.1867	-1.32	0.8092	-0.0308	0.0233
rs1339227			11878	0.761	0.304	0.3287	0.0059	0.0195
rs6466055	rs2057883	0.932	14024	0.1272	1.525	0.3526	0.0270	0.0177
rs211829			14015	0.1707	1.37	0.6271	0.0239	0.0175
rs13240464	rs38752	1	16115	0.8176	0.231	0.6543	0.0038	0.0166
rs7801375			14046	0.5881	-0.542	0.1575	-0.0126	0.0232
rs3735025	rs12540417	1	14024	0.4342	0.782	0.6308	0.0137	0.0175
rs10503253			16121	0.5243	0.637	0.1928	0.0127	0.0200
rs6984242	rs867743	0.962	16123	0.2244	-1.215	0.5882	-0.0194	0.0160

rs36068923	rs13267290	0.943	16124	0.1943	-1.298	0.7925	-0.0252	0.0194
rs4129585			16112	0.2105	-1.252	0.4359	-0.0199	0.0159
rs11139497			11853	0.09111	-1.69	0.3204	-0.0333	0.0197
rs7893279			16118	0.2145	-1.241	0.8846	-0.0306	0.0247
rs55833108	rs2274341	0.955	14017	0.2873	1.064	0.2332	0.0213	0.0200
rs11027857			13940	0.728	-0.348	0.5203	-0.0059	0.0170
rs9420			16123	0.2872	-1.064	0.3228	-0.0179	0.0168
rs12421382			11060	0.7766	0.284	0.3138	0.0058	0.0205
rs2514218			11844	0.5229	-0.639	0.3595	-0.0122	0.0191
rs77502336	rs7927176	0.957	13968	0.02921	2.181	0.3257	0.0394	0.0181
rs10791097			11895	0.9085	0.115	0.4802	0.0021	0.0184
rs75059851	rs3802924	1	13969	0.681	-0.411	0.8038	-0.0088	0.0213
rs2007044			16122	0.9827	0.022	0.619	0.0004	0.0162
rs2239063			11830	0.07028	1.81	0.7154	0.0369	0.0204
rs679087	rs302321	1	16122	0.01359	-2.468	0.3485	-0.0408	0.0165
rs4240748			11859	0.8458	0.195	0.3738	0.0037	0.0190
rs10860964			14032	0.3704	-0.896	0.6265	-0.0156	0.0175
rs2851447			11891	0.2399	-1.175	0.7615	-0.0253	0.0215
rs2068012			11854	0.361	0.913	0.7717	0.0200	0.0219
rs2693698			16123	0.0873	-1.71	0.4564	-0.0270	0.0158
rs12903146	rs2414716	0.967	16123	0.6669	-0.43	0.5431	-0.0068	0.0158
rs12148337			13991	0.567	-0.572	0.4545	-0.0097	0.0170
rs8042374			16069	0.733	-0.341	0.7707	-0.0064	0.0188
rs950169			16116	0.2078	1.26	0.2777	0.0222	0.0176
rs4702			16122	0.5442	-0.607	0.5822	-0.0097	0.0160
rs9922678			13974	0.4272	-0.794	0.2923	-0.0148	0.0186
rs7405404	rs7185124	1	16087	0.5022	0.671	0.2302	0.0126	0.0187
rs12325245			13912	0.5584	-0.585	0.8391	-0.0135	0.0231
rs8044995			14043	0.6047	0.518	0.1641	0.0118	0.0228
rs4523957			11819	0.2791	1.082	0.6263	0.0206	0.0190
rs78322266	rs17594721	1	16122	0.5205	0.643	0.0324	0.0286	0.0445
rs9636107			13988	0.5145	0.652	0.5205	0.0110	0.0169
rs72934570	rs9945732	1	16119	0.3333	-0.968	0.0629	-0.0314	0.0324
rs715170			13982	0.9896	-0.013	0.2682	-0.0002	0.0191
rs2905426	rs1469712	1	14039	0.7268	0.349	0.6426	0.0061	0.0176
rs2053079			13993	0.4972	0.679	0.7538	0.0133	0.0196
rs56873913	rs10417980	0.953	4735	0.9481	-0.065	0.763	-0.0022	0.0342
rs6065094			16119	0.9319	0.086	0.3341	0.0014	0.0167
rs7267348			14000	0.1677	-1.38	0.756	-0.0272	0.0197
rs1023500			13984	0.795	-0.26	0.8176	-0.0057	0.0219

1. Proxy used in SUNLIGHT; 2. R-squared value indicating linkage disequilibrium between variant in PGC and proxy; 3. Z-value from association test; 4. Effect allele frequency (EAF) corresponds to effect allele from Table S3; 5. Constructed beta value. Calculated as Beta=z-score/sqrt(N) * 1/SQRT(EAF(1-EAF)). This calculation assumes that the standard errors are proportional to the inverse-square root of the sample size multiplied by the variance of the genetic variant as a random variable (variance = EAF(1-EAF)). This result should hold asymptotically; 6. Constructed standard error. Calculated as Beta/z-score.

Table S5. Results from the fixed effects meta-analysis, likelihood approach and Egger

	Beta[1]	95% CI	P-value
Fixed effects meta-analysis	0.051	(-0.006, 0.108)	0.08
Likelihood Approach	0.057	(-0.001, 0.114)	0.05
Egger regression- Intercept	-0.003	(-0.026,0.019)	0.79
Egger regression- Slope	0.096	(-0.209, 0.401)	0.54

1. Beta coefficients are constructed from z scores. Effect sizes are not interpretable.

Figure S1. Mendelian randomization analysis of the effect of 25(OH)D on schizophrenia using SNPs related to 25(OH)D synthesis

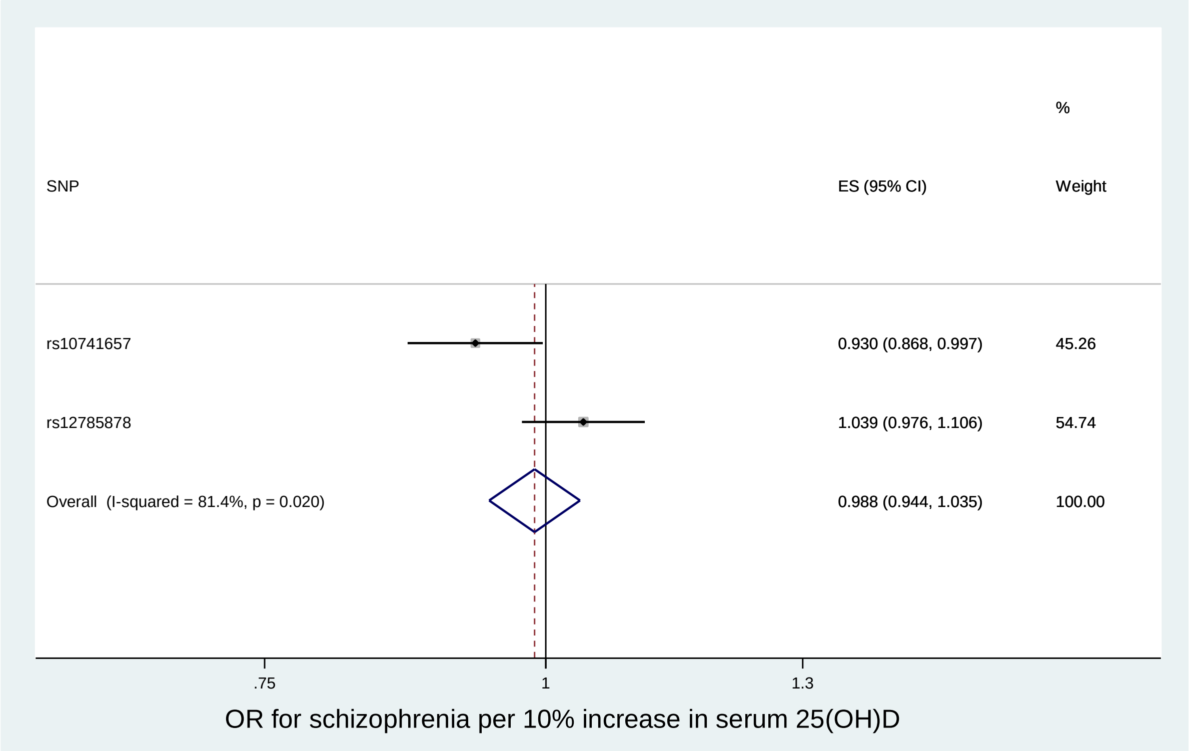

Results combined in inverse variance weighted fixed effects meta-analysis. The result from the likelihood approach was similar (OR: 0.987, 95% CI: 0.943, 1.034).

Figure S2. Mendelian randomization analysis of the effect of 25(OH)D on schizophrenia using SNPs related to 25(OH)D metabolism

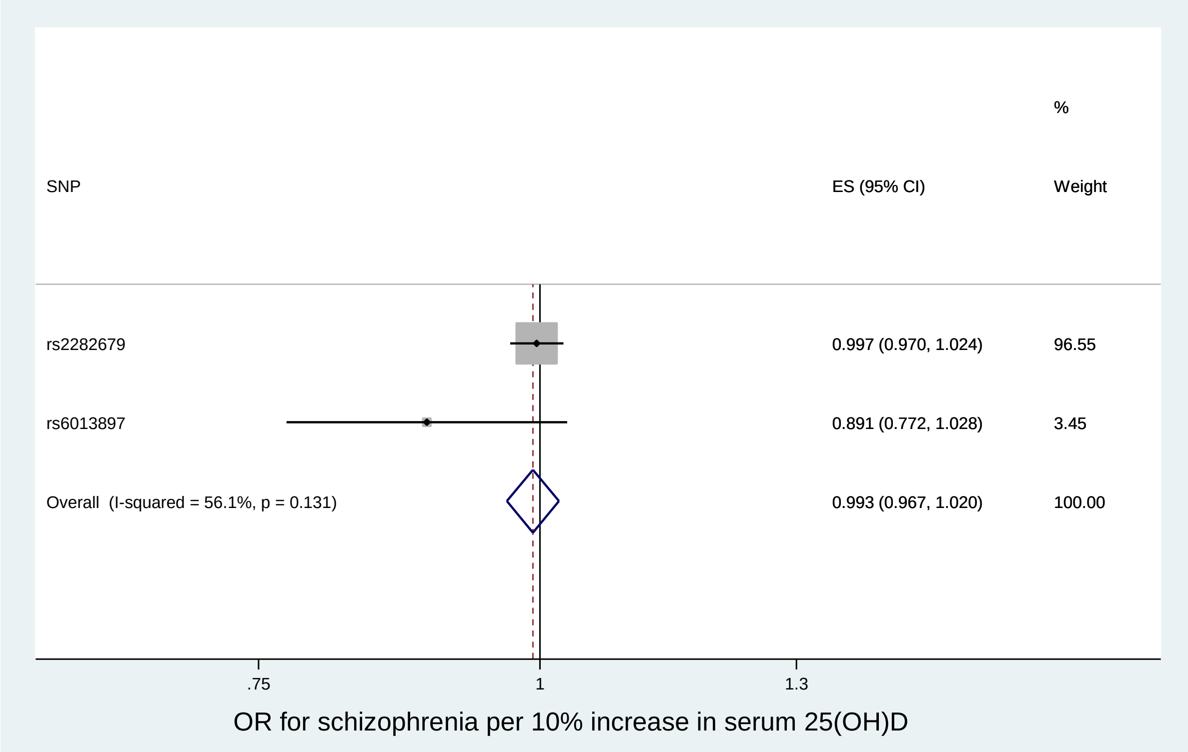

Results combined in inverse variance weighted fixed effects meta-analysis. The result from the likelihood approach was similar (OR: 0.993, 95% CI: 0.966, 1.019).

Figure S3. Mendelian randomization analysis of the effect of 25(OH)D on schizophrenia excluding the *GC* SNP

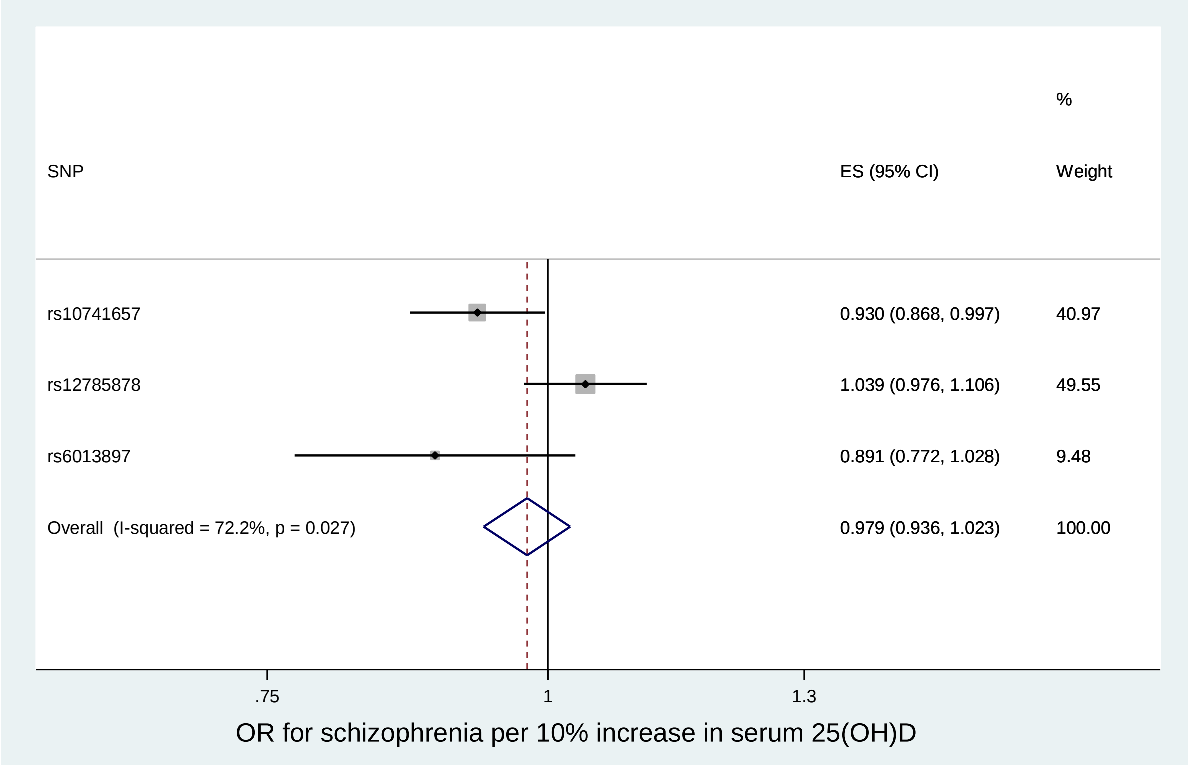

Results combined in inverse variance weighted fixed effects meta-analysis. The result from the likelihood approach was similar (OR: 0.977, 95% CI: 0.935, 1.021).

Figure S4. Mendelian randomization analysis of the effect of 25(OH)D on schizophrenia excluding the *DHCR7* SNP

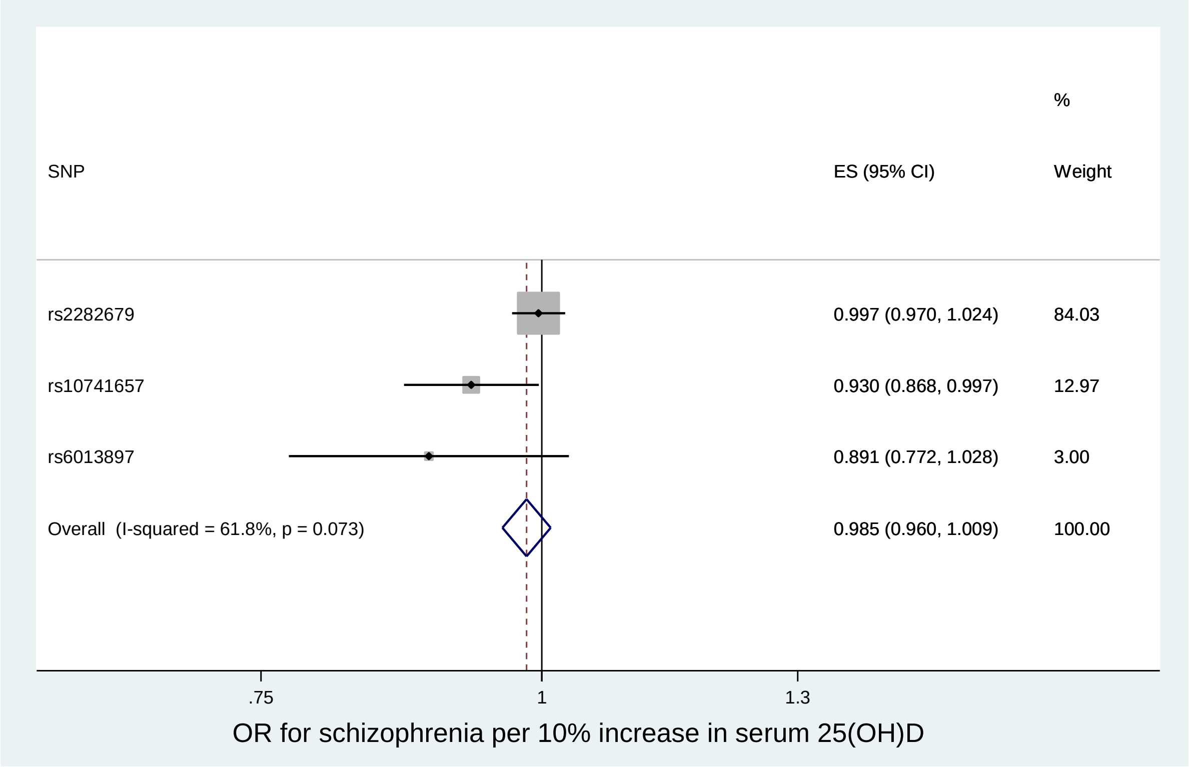

Results combined in inverse variance weighted fixed effects meta-analysis. The result from the likelihood approach was similar (OR: 0.984, 95% CI: 0.960, 1.009).
